# The ups and downs of human UNC93B1: Autoimmune and viral conditions

**DOI:** 10.70962/jhi.20260059

**Published:** 2026-10-20

**Authors:** Elina Idiiatullina, Jean-Laurent Casanova, Shen-Ying Zhang

**Affiliations:** 1 St. Giles Laboratory of Human Genetics of Infectious Diseases, Rockefeller Branch, Rockefeller University, New York, NY, USA; 2 Laboratory of Human Genetics of Infectious Diseases, Dallas Branch, University of Texas Southwestern, Dallas, TX, USA; 3 Center for the Genetics of Host Defense, The University of Texas Southwestern Medical Center, Dallas, TX, USA; 4 Université Paris Cité, Institut Imagine, Laboratory of Human Genetics of Infectious Diseases, Necker Branch, INSERM UMR1163, Paris, France; 5 Service de Pédiatrie, AP-HP, Hôpital Necker-Enfants Malades, Paris, France; 6 Department of Pediatrics, University of Texas Southwestern, Dallas, TX, USA; 7 Department of Immunology, University of Texas Southwestern, Dallas, TX, USA; 8 Howard Hughes Medical Institute, New York, NY, USA; 9 Children’s Hospital Research Institute, Dallas, TX, USA; 10 Department of Neuroscience, University of Texas Southwestern, Dallas, TX, USA; 11 Department of Neurology, University of Texas Southwestern, Dallas, TX, USA

## Abstract

Mouse Unc93b1 was identified in 2006 by forward genetics as an ER membrane protein essential for the activity of the endosomal nucleic acid–sensing receptors TLR3, TLR7, and TLR9. In vitro studies in mouse and human cells have revealed its cellular functions, including ER-based stabilization and trafficking of endosomal TLR proteins (TLR3, TLR7, TLR8, and TLR9) and regulation of signaling thresholds through competitive trafficking control (TLR7 and TLR9). Human genetic studies have clarified the physiological role of UNC93B1 in natural conditions. Biallelic loss-of-function *UNC93B1* mutations impair TLR3 signaling in central nervous system–resident cells, thereby conferring a predisposition to HSV-1 encephalitis. UNC93B1 deficiency may also predispose to other viral infections, as suggested by patients with inherited TLR3 or TLR7 deficiency. Conversely, monoallelic or biallelic *UNC93B1* gain-of-function variants enhancing TLR7 and/or TLR8 signaling predispose patients to autoimmunity and inflammation, including early-onset systemic lupus erythematosus and chilblain lupus. Thus, in humans, UNC93B1 maintains a delicate balance between host defense and tolerance to self.

## Teaser

Biallelic loss-of-function UNC93B1 mutations impair TLR3 signaling in central nervous system–resident cells, underlying herpes simplex virus 1 encephalitis. Conversely, monoallelic or biallelic gain-of-function UNC93B1 mutations enhance TLR7 and/or TLR8 signaling, driving autoimmunity. Thus, in humans, UNC93B1 maintains a delicate balance between host defense and tolerance to self.

## Introduction

The vertebrate UNC93B1 was named after its ortholog in *Caenorhabditis elegans*, unc-93, which encodes a regulator of a two-pore potassium channel complex involved in muscle contraction. Mutations in unc-93 cause an uncoordinated (UNC) phenotype (1). In 2006, UNC93B1 was shown to be an ER membrane protein essential for the activity of endosomal nucleic acid–sensing TLRs in mice and humans, following parallel research into the genetic cause of the mouse triple D (“3d”) phenotype, consisting of defective TLR3, TLR7, and TLR9 signaling (2), and the cause of defective TLR3, TLR7, TLR8, and TLR9 signaling in rare children with HSV-1 encephalitis (HSE) (3). In mice, the 3d phenotype was identified through ENU mutagenesis and forward genetic screening. These mice were homozygous for a missense mutation (H412R) of *Unc93b1* that abolished signaling via TLR3, TLR7, and TLR9, receptors responsible for detecting double-stranded RNA, single-stranded RNA, and unmethylated CpG DNA, respectively (2). As a result, 3d mice displayed profound deficits of type I IFN production and were highly susceptible to murine cytomegalovirus infection (2). The discovery of the causal role of Unc93b1 in the 3d mouse phenotype was followed by the identification of biallelic loss-of-function (LOF) frameshift or essential splice-site mutations in human *UNC93B1* in two otherwise healthy children with HSE, with abolished TLR3 responses in fibroblasts and impaired TLR7, TLR8, and TLR9 responses in leukocytes (3). The identification of UNC93B1 as a critical regulator of mammalian innate immunity was unexpected because its *C*. *elegans* ortholog had previously been implicated only in neuromuscular function (1). Here, we review recent advances in our understanding of the physiological roles of UNC93B1 in humans, highlighting the insights provided by human genetics.

### Molecular and cell biology of UNC93B1

*UNC93B1* is evolutionarily conserved from invertebrates to mammals (2, 4, 5, 6, 7). Mammalian and invertebrate UNC93B1 proteins have similar structural organization and membrane topology despite their modest sequence identity. In humans, *UNC93B1* encodes a 597–amino acid multi-pass membrane protein containing 12 transmembrane helices, with cytosolic N and C termini. Mouse *Unc93b1* encodes a protein of similar size and topology, reflecting strong structural conservation between the two species (2). The protein resides within intracellular membranes, primarily the ER, but is also present in ER-Golgi intermediate compartments, the Golgi apparatus, and endosomal vesicles during trafficking. It contains luminal and cytosolic loops that mediate protein–protein interactions. Structure–function analyses in mice established the importance of the conserved transmembrane residues, as illustrated by the H412R mutation in the 3d mouse model, which disrupts normal protein function without abolishing the production of the protein (2). The orthologs of the various vertebrate species have a similar membrane topology and conserved functional domains, supporting the idea that UNC93B1 evolved early as a multi-pass ER-resident membrane protein with conserved biochemical properties (6, 7). Cryo-electron microscopy studies subsequently resolved the structures of the human TLR7–UNC93B1 and human and mouse TLR3–UNC93B1 complexes, revealing extensive interactions between the amino-terminal six-helix bundle of UNC93B1 and the transmembrane and luminal juxtamembrane regions of the TLRs, providing a structural framework for understanding receptor trafficking and signaling regulation (8). UNC93B1 is broadly expressed across human tissues from both hematopoietic and non-hematopoietic compartments, and its levels are highest in professional antigen-presenting cells, including dendritic cells, monocytes, macrophages, and B cells (9). The levels of this protein are particularly abundant in dendritic cells and macrophages in mice and humans (10, 11). Type I IFNs upregulate *UNC93B1* transcription (12, 13). Together, these findings show that UNC93B1 is a widely expressed, mostly ER-resident membrane protein, the abundance of which is modulated by cytokine signals.

At steady state, the human and mouse UNC93B1 proteins are located predominantly in the ER, where they reside as integral membrane proteins (2, 10, 11, 14). UNC93B1 is co-translationally inserted into ER membranes, with which it remains associated during its subsequent trafficking through the secretory pathway (15). Proteomic and imaging studies have shown that UNC93B1 remains associated with its known client proteins after exiting the ER and that it accompanies these proteins in the post-Golgi compartments (15, 16). Functionally, both mouse and human UNC93B1 control the trafficking, stability, and activation of endosomal TLR3, TLR7, TLR8, and TLR9. Murine TLR8 is much less responsive to classical agonists than human TLR8. This initially led to its being considered nonfunctional, but there is growing evidence to suggest that murine TLR8 can signal in myeloid cells in response to specific endogenous and synthetic ligands, with recent studies further expanding the repertoire of candidate murine TLR8 ligands (17, 18, 19, 20, 21, *Preprint*). These receptors detect double-stranded or single-stranded nucleic acids in endolysosomes, and signal via TRIF (TLR3) or MyD88 (TLR7/8/9) (4, 22, 23, 24). In the ER, UNC93B1 binds the transmembrane domains of newly synthesized TLR3, TLR7, TLR8, and TLR9, promoting their incorporation into COPII-coated vesicles, thereby facilitating their export from the ER (2, 4, 15, 22). UNC93B1 stabilizes these receptors and prevents their premature degradation, thereby regulating receptor abundance independently of transcription (11). After exiting the ER, UNC93B1 remains associated with TLRs and participates in their post-Golgi trafficking through various pathways. TLR9 trafficking follows an AP-2–dependent plasma membrane route to the endolysosomes. TLR7 associates with AP-4 in these organelles and is thought to be trafficked directly from the trans-Golgi network to late endosomes (15). Recent studies have suggested that the BORC–Arl8b complex regulates later stages of TLR7-containing UNC93B1 complex trafficking and degradative sorting (25). UNC93B1–TLR complexes are differentially regulated within endosomal compartments. In mice, UNC93B1 also regulates the relative extents to which TLR7 and TLR9 undergo trafficking through competitive binding and transport mechanisms, thereby maintaining the balance between RNA- and DNA-sensing TLR responses (26). TLR3 and TLR9 undergo regulated release from UNC93B1 specifically within endosomes, a step required for ligand binding and signal transduction that reinforces compartmentalized receptor activation. By contrast, TLR7 remains associated with UNC93B1 in endosomes and is controlled by different regulatory mechanisms (16). UNC93B1 also recruits syntenin-1 to regulate TLR7 signaling, thereby constraining receptor activation in vivo (27). N-glycosylation at Asn272 selectively facilitates the CpG-induced conformational changes required for TLR9 activation and subsequent MyD88 recruitment, further refining signaling output (28). The mouse UNC93B1 has also been reported to be required for TLR5 plasma membrane localization and flagellin-induced signaling, extending its role beyond endolysosomal TLRs (5). In mice, UNC93B1 also regulates TLR11, TLR12, and TLR13—receptors not present in humans (15).

Beyond TLRs, the human and mouse UNC93B1 proteins regulate additional immune signaling pathways through interactions with non-TLR client proteins. Early studies suggested that UNC93B1 contributed to MHC class II antigen presentation and antigen cross-presentation. However, subsequent studies were unable to reproduce the antigen-presentation defects originally reported in 3d mice, raising questions as to whether these findings reflected an intrinsic role of UNC93B1 in antigen presentation or were secondary to alterations of the endosomal organization and trafficking of other molecules involved in MHC antigen presentation. The contribution of UNC93B1 to antigen presentation therefore remains unclear and may depend on experimental context (2, 29). It has recently been shown that, in human and mouse dendritic cells, UNC93B1 interacts with the ER calcium sensor STIM1, keeping STIM1 in a dimeric state primed for translocation to ER–plasma membrane junctions, thereby enhancing store-operated calcium entry, as demonstrated by the impaired calcium flux and defective STIM1 puncta formation and antigen cross-presentation observed in UNC93B1-deficient dendritic cells (30, 31). UNC93B1 also modulates cytosolic nucleic-acid sensing by promoting STING degradation via lysosomal pathways, thereby decreasing cGAS–STING-mediated IFN responses (32, 33). This regulatory function highlights the role of this protein in maintaining the homeostasis of intracellular DNA sensing. By contrast, in other animal models, such as renal injury models in rodents, UNC93B1 enhances cGAS–STING pathway activation, contributing to oxidative damage and ER stress; the inhibition of UNC93B1 reduces STING signaling and attenuates tissue damage (34). These findings suggest that UNC93B1 is an important coordinator at the crossroads of TLR- and non-TLR–mediated cellular responses in diverse biological contexts, the TLR-related functions of UNC93B1 being the best characterized to date.

### Autosomal recessive (AR) UNC93B1 LOF underlies HSE via TLR3

**Figure 1.**
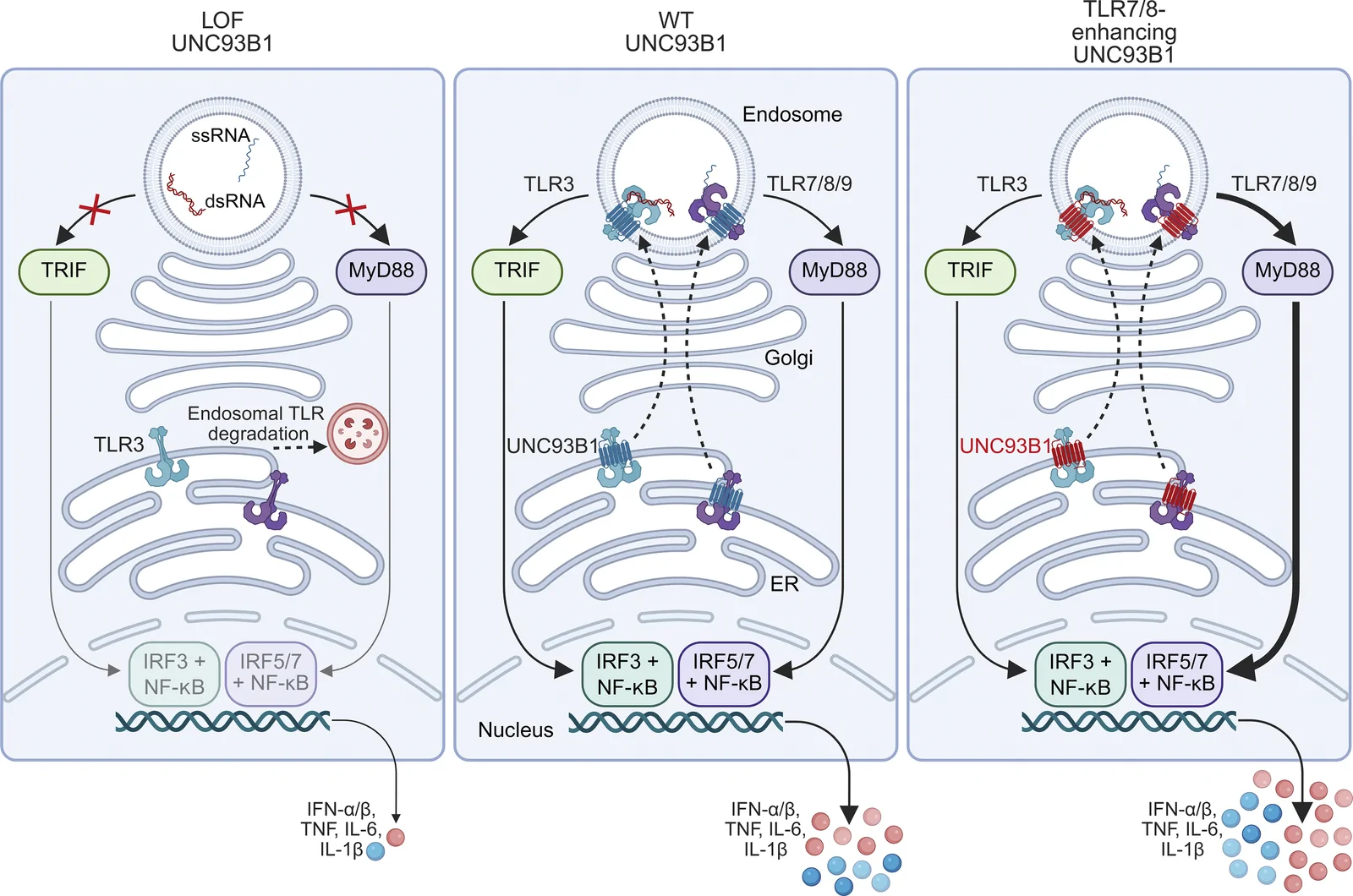
**Consequences of LOF and signaling-enhancing UNC93B1 variants on endosomal TLR signaling and human disease.** UNC93B1 regulates the trafficking and signaling of multiple endosomal TLRs. Middle panel: UNC93B1 resides in the ER and escorts TLR3 and TLR7/8/9 to endosomes. Upon ligand recognition (double-stranded [ds] RNA for TLR3, single-stranded [ss] RNA for TLR7/8, and CpG DNA for TLR9), TLR3 signals via TRIF to activate IRF3 and NF-κB, whereas TLR7/8/9 signal via MyD88 and IRAK4 to activate IRF5, IRF7, and NF-κB. The coordinated activation of these transcription factors induces the expression of type I IFNs and other cytokines. Left panel: In the absence of functional UNC93B1, the trafficking of endosomal TLRs from the ER to endosomes is impaired, resulting in receptor retention and degradation. Consequently, TLR3 fails to sense dsRNA in endosomes, leading to defective type I IFN–mediated antiviral immunity and susceptibility to HSE. Right panel: UNC93B1 variants associated with enhanced TLR7 and/or TLR8 signaling promote exaggerated downstream responses to ssRNA. This results in excessive levels of type I IFNs and pro-inflammatory cytokines (e.g., TNF, IL-1, and IL-6), driving autoinflammatory and autoimmune disease.

In 2006, AR UNC93B1 deficiency was identified as the first monogenic cause of HSE in otherwise healthy children (3, 24, 35, 36) (Fig. 1, Table 1). Biallelic LOF frameshift or splice-site mutations impair UNC93B1 expression, causing a complete loss of TLR3-dependent responses in fibroblasts and impaired TLR7-, TLR8-, and TLR9-dependent cellular responses in leukocytes (3, 24). Subsequent studies of patients with other inherited defects affecting TLR3 signaling provided additional support for a nonredundant role of TLR3-dependent type I IFN immunity in protection against HSV-1 within the CNS (3, 37, 38, 39). Patients with defects of this pathway typically display preserved systemic immunity, including normal lymphocyte subsets, immunoglobulins, and vaccine responses, but remain selectively vulnerable to HSE, highlighting the tissue-specific requirement for TLR3-dependent antiviral defense (3, 23, 24, 37, 40). Incomplete penetrance has been observed, with mutation carriers infected with HSV-1 but remaining asymptomatic, suggesting that additional genetic, epigenetic, or environmental factors influence disease expression (24, 41, 42, 43).

**Table 1.** Human UNC93B1 variants associated with viral susceptibility or inflammatory disease

Variant	Zygosity	Inheritance	Penetrance	Molecular mechanism	Clinical phenotype	Patient sex	No. of patients/no. of families	Reference
**A. Biallelic LOF variants**								
c.1034_1037del p.Phe345fs (F345fs)	Homozygous	AR	Complete	Impaired TLR3/7/8/9-mediated type I/III IFN induction	Childhood HSE	1 M	1/1	(3, 40)
c.781G>A p.Arg261His (R261H)	Homozygous	AR	Incomplete^c^	Impaired TLR3/7/8/9-mediated type I/III IFN induction	Childhood HSE	1 F	1 + 1/1^d^	(3, 40)
**B. Monoallelic LOF variants**								
UNC93B1: c.1210C>T; p.Pro404Ser (P404S) IRF7: c.338G>C; p.Arg113Pro (R113P)	Heterozygous	Unknown (parents not tested; possible combined contribution with a heterozygous IRF7 variant)	n/a (single case; combined variants)	Lower levels of binding to TLR3 and impaired poly(I:C)-induced type I IFN production in vitro	Neonatal HSV-1 SEM disease; HSE at 1 year of age	1 M	1/1	(44)
p.Glu96* (E96*)^a^	Heterozygous	Presumed AD^a^	Unknown	Impaired TLR3-dependent IFN induction	Hypoxemic COVID-19 pneumonia	n/r	1/1	(45)
Multiple rare monoallelic UNC93B1 variants: p.Ser164Leu (S164L), p.Ala361Thr (A361T), p.Val379Met (V379M), p.Ala385Thr (A385T), p.Val402Leu (V402L), p.Tyr435Cys (Y435C), p.Gly553Asp (G553D), p.Gly592Arg (G592R)	Heterozygous	Presumed AD	Incomplete	Impairing TLR3-dependent IFN induction	Hypoxemic COVID-19 pneumonia	n/r	10/10	(45)
**C. Variants associated with enhanced TLR7/TLR8 signaling**								
p.Glu92Gly (E92G)	Homozygous	AR	Complete	UNC93B1 instability with lower levels of UNC93B1–TLR7 interaction causing selective TLR7 (±TLR8) hyperactivation and constitutive type I IFN signaling	Very early-onset SLE with a strong IFN signature; systemic autoimmunity	1 M, 1 F	2/1	(46)
p.Arg336Leu (R336L)	Heterozygous	AD	Unknown	TM6–TM7 helix variant promoting TLR7 (±TLR8) hyperactivation with enhanced cytokine and IFN signatures	Childhood-onset SLE with lymphoproliferation (ALPS-like features)	2 M	2/1	(46)
p.Thr93Ile (T93I)	Heterozygous	AD	Unknown	Lower levels of UNC93B1–TLR7 interaction with an increase in TLR7/8-ligand responsiveness	Early-onset cutaneous tumid lupus	2 M, 2 F	4/1	(47)
p.Arg336Cys (R336C)	Heterozygous (de novo^b^)	AD	Unknown	Enhanced K63 ubiquitylation and association with syntenin-1, resulting in dysregulation of TLR7/8 signal termination and enhanced responses	Juvenile idiopathic arthritis, neuroinflammatory movement disorder, tumid/chilblain lupus; IFN-high	1 F	1/1	(47)
p.Thr314Ala (T314A)	Heterozygous	AD	Unknown	TM6–TM7 helix variant causing TLR7/8 hyperresponsiveness with an increase in basal IFN and NF-κB signaling	Childhood-onset SLE with lupus nephritis	1 F	1/1	(48)
p.Val117Leu (V117L)	Heterozygous	AD	n/a (risk allele)	Protomer-interface variant causing selective TLR7/8 hyperresponsiveness with increases in basal IFN and NF-κB programs	Childhood-onset SLE	7 F	7/7	(48)
p.Ile317Met (I317M)	Homozygous	Presumed AR	Unknown	Increase in TLR7 signaling with weaker TLR8 activation	Childhood-onset SLE with severe autoimmune hemolytic anemia	1 F	1/1	(49)
p.Gly325Cys (G325C)	Heterozygous	AD	Incomplete	Increases in TLR7 and TLR8 signaling with a strong IFN signature and enhanced UNC93B1–TLR8 association	Early-onset SLE with pulmonary arterial hypertension	1 F	1 + 1/1	(49)
p.Leu330Arg (L330R)	Heterozygous	Presumed AD	Unknown	Enhanced UNC93B1–TLR8 interaction; TLR8-biased signaling enhancement with an increase in NF-κB signaling in vitro	Chilblain lupus	1 M	1/1	(49)
p.Arg466Ser (R466S)	Heterozygous (de novo)	AD	Unknown	Enhanced UNC93B1–TLR8 interaction; TLR8-biased signaling enhancement in vitro	Chilblain lupus	1 F	1/1	(49)
p.Arg525Pro (R525P)	Heterozygous	AD	Unknown	Enhanced TLR8 signaling in vitro, with exaggerated ex vivo monocyte responses to TLR8 agonists	Familial chilblain lupus	2 M, 2 F	4/1	(49)
p.Arg336His (R336H)	Heterozygous	AD	Unknown	Probably pathogenic: no direct functional validation	Juvenile arthritis/rheumatoid arthritis spectrum ± ITP; prominent interstitial pneumonia	2 M, 2 F	4/1	(50)
p.Glu49dup (E49dup; “6xE”)	Heterozygous	AD	Incomplete	Lower levels of UNC93B1–ARL8B/BORC interaction impair BORC-mediated degradative sorting of TLR7, resulting in endosomal TLR7 accumulation and enhanced signaling	Childhood-onset SLE with systemic autoimmunity	1 F	1 + 1/1	(25)

AD, autosomal dominant; ALPS, autoimmune lymphoproliferative syndrome; ITP, immune thrombocytopenia; SEM, skin/eye/mouth.

a Presumed AD/AR: inheritance inferred from zygosity, segregation, recurrence within the family, and/or functional data, but not definitively established.

b De novo: variant absent from both parents when parental test results were available.

c Incomplete penetrance: at least one carrier was clinically unaffected or substantially less severely affected than other carriers.

d 1 + 1/1: one affected carrier and one unaffected carrier within the same family.

Studies using patient-derived induced pluripotent stem cell (iPSC) models subsequently established the cellular basis of HSE susceptibility in UNC93B1 deficiency (40). UNC93B1-deficient cortical neurons and oligodendrocytes display impaired IFN production and are highly susceptible to HSV-1 infection, consistent with a nonredundant role for TLR3–UNC93B1 signaling in the intrinsic antiviral immunity of selected CNS cell populations (40). The restoration of wild-type UNC93B1 rescued antiviral responses in patient-derived cortical neurons, providing additional support for causality (40). By contrast, iPSC-derived TLR3-deficient trigeminal neurons were not found to be highly susceptible to HSV-1 infection (51). TLR3 was later shown to control constitutive type I IFN production in fibroblasts and cortical neurons, suggesting that deficiencies of the TLR3–IFN axis may confer a predisposition to HSE not through impaired viral sensing, but through a lower baseline antiviral readiness of CNS-resident cells (52). Collectively, these studies established TLR3–UNC93B1-dependent IFN immunity as a non-redundant antiviral pathway in the CNS, redefining HSE as a tissue-specific infectious disease caused by defects of brain cell–intrinsic antiviral defense.

Patients with X-linked recessive TLR7 deficiency are highly susceptible to life-threatening COVID-19 pneumonia (53, 54). Patients with UNC93B1 deficiency are likely to be similarly vulnerable, as shown for patients with IRAK4 or MyD88 deficiency, whose cells do not respond to TLR7 stimulation (55). No patients with inherited deficiencies of TLR8 or TLR9 have been reported to date. Individuals with such disorders may be susceptible to viral infections that remain unidentified due to limited pathogen exposure, incomplete penetrance, or underdiagnosis. Remarkably, despite the discovery of UNC93B1 deficiency almost two decades ago, only two patients with complete biallelic deficiency have been reported to date (3, 40). Consistent with the extreme rarity of complete UNC93B1 deficiency, no homozygous predicted LOF variants are reported in gnomAD, which includes exome and genome sequencing data for more than 800,000 individuals from the general population. The two patients with AR UNC93B1 deficiency were identified from our cohort of about 520 patients with HSE, further illustrating the rarity of this genetic etiology. This apparent rarity may reflect extremely low allele frequencies, incomplete penetrance, underdiagnosis, a redundancy of UNC93B1-dependent pathways outside specific tissues, or limited exposure to relevant pathogens. Additional longitudinal studies and broader genetic screening may help to refine the prevalence and phenotypic spectrum of complete UNC93B1 deficiency.

### Monoallelic UNC93B1 variants in severe viral disease

Relatives of the HSE patients with AR UNC93B1 deficiency heterozygous for the same LOF variant did not develop the same disease, confirming the recessive mode of inheritance of predisposition to HSE. However, it remains unclear whether monoallelic UNC93B1 variants confer a predisposition to viral diseases other than HSE. Heterozygosity for rare experimentally LOF UNC93B1 variants has been reported to be overrepresented in patients with severe COVID-19 pneumonia (Table 1) (45). It remains unclear whether some of these variants act in a dominant-negative manner. Moreover, no cellular studies have been performed on cells from variant carriers to clarify the impact of heterozygosity for these variants on UNC93B1-dependent pathways. Thus, by contrast to the recessive deficiencies affecting TLR3-, TLR7-, MyD88-, or IRAK4-dependent pathways (52, 53, 55, 56, 57, 58), the contribution of these monoallelic variants to viral disease susceptibility has yet to be investigated. An infant with neonatal HSV-1 skin/eye/mouth disease who later developed HSE at the age of 1 year was recently found to be heterozygous for missense variants of *UNC93B1* and *IRF7*. Both the UNC93B1 and IRF7 variants were LOF in vitro, and the patient’s peripheral blood mononuclear cells (PBMCs) had impaired responses to poly(I:C) stimulation. Functional studies in THP-1 monocytic cells and human fibroblasts expressing the variants demonstrated an impairment of TLR3-dependent type I IFN production. Moreover, fibroblasts expressing the variants had impaired type I IFN responses and higher intracellular viral DNA levels following HSV-1 infection. These data suggest a possible oligogenic contribution to disease susceptibility but further molecular validation is required (44).

### UNC93B1 variants enhancing TLR7 and/or TLR8 signaling confer a predisposition to autoimmunity and inflammation

**Figure 2.**
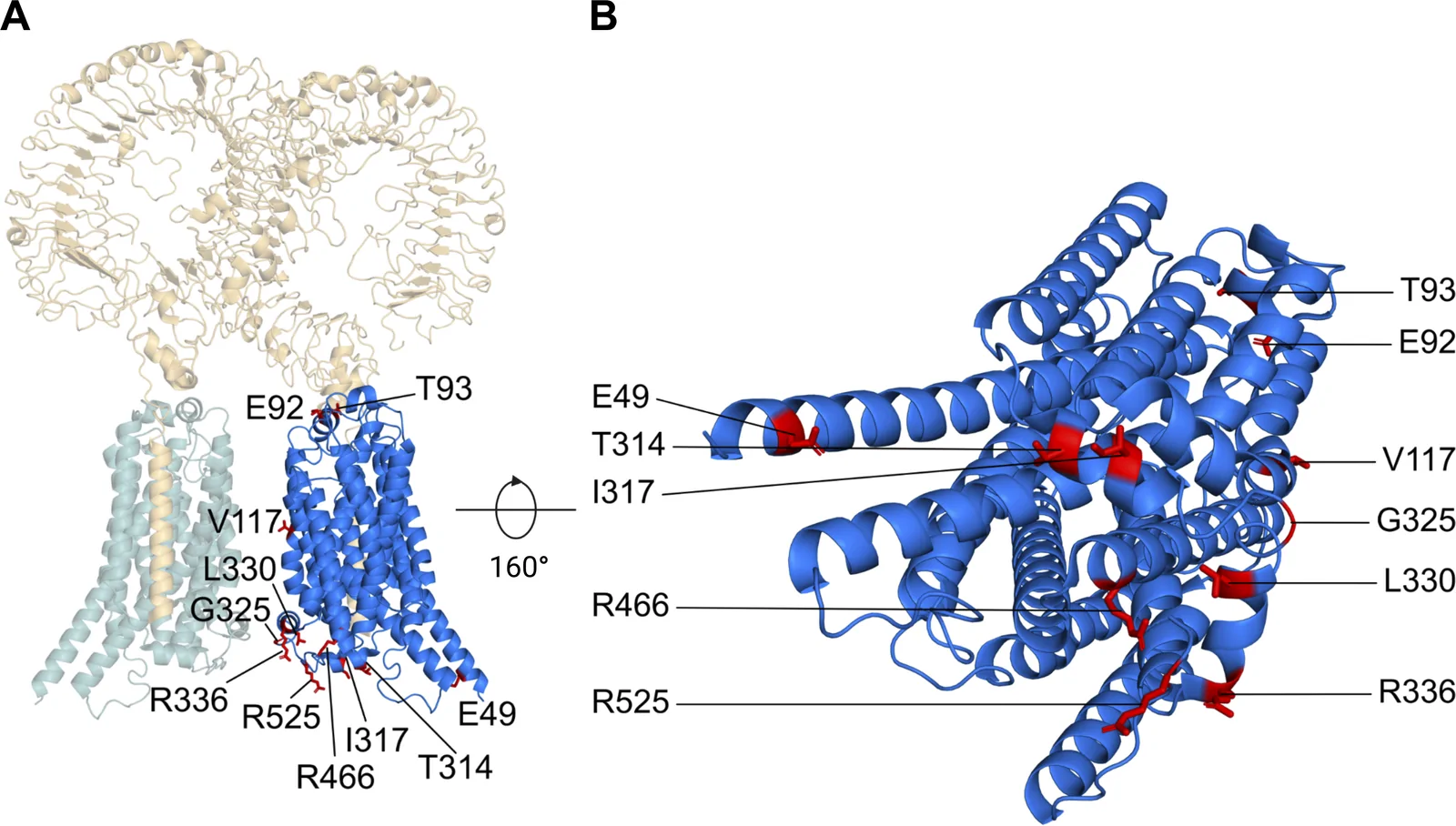
**Mapping of autoimmune and inflammatory disease–associated UNC93B1 variants onto the human TLR7–UNC93B1 complex.** Structural representation based on the cryo-EM structure of the human TLR7–UNC93B1 complex reported by Ishida et al. (8). **(A)** Overall structure showing the positions of disease-associated UNC93B1 residues reported to date. **(B)** Rotated view of UNC93B1 highlighting these residues. Disease-associated residues are shown in red.

In 2024, heterozygosity or homozygosity for *UNC93B1* variants associated with enhanced TLR7 and/or TLR8 signaling was reported in patients with systemic lupus erythematosus (SLE), chilblain lupus, and related inflammatory phenotypes (25, 46, 47, 48, 49, 59). These disease-associated variants include missense mutations (E92G, T93I, V117L, T314A, I317M, G325C, L330R, R336C, R336L, R336H, R466S, and R525P) and the E49dup insertion (47, 48, 49) (Table 1). Most affected individuals reported to date were heterozygous, whereas homozygosity for I317M has been reported in a single patient, and homozygosity for E92G in two affected siblings from a consanguineous family (46, 49). E92G appears to have a gene-dosage effect, with whole-blood cells from heterozygous carriers displaying intermediate TLR7 hyperresponsiveness in vitro, whereas cells from homozygous individuals display markedly greater TLR7 responsiveness corresponding to the more severe clinical phenotype (46). These variants cluster in luminal, transmembrane, and cytoplasmic regions of UNC93B1 and have mechanistically different effects on the protein (Fig. 2). Some of the mutations alter protein stability or receptor interaction, as illustrated by E92G, which destabilizes UNC93B1 and reduces UNC93B1–TLR7 interaction while increasing downstream TLR7 signaling (46). Variants affecting residue R336 (R336C, R336L, and R336H) are located within a cytoplasmic regulatory region implicated in limiting TLR7 signaling and promoting signal termination. Functional studies suggest that substitutions at this residue dysregulate TLR7 responses, although the underlying molecular mechanisms remain incompletely understood (27, 46, 47, 50). Other variants located in transmembrane or protomer-interface regions (T314A, V117L, and T93I) may alter receptor handling or conformational dynamics without uniformly destabilizing the protein, thereby enhancing downstream TLR7 and/or TLR8 signaling (47, 48). E49dup acts via another mechanism, weakening the recruitment of the BLOC-1–related complex (BORC)–Arl8b complex responsible for regulating the degradative sorting of TLR7-containing UNC93B1 complexes, thereby prolonging the residence of TLR7 in signaling-competent endosomal compartments (25). Consistent with the importance of restraining TLR7 signaling, preferential trafficking of TLR7 at the expense of TLR9 has been shown to result in excessive TLR7 signaling and systemic inflammation in mice (26). Thus, although these variants are commonly described as “gain-of-function” (GOF), this terminology primarily reflects enhanced signaling downstream from TLR7 and/or TLR8 rather than a uniform enhancement of all UNC93B1 activities. Some variants can be designated GOF-TLR7 (e.g., E49dup), GOF-TLR8 (e.g., R525P), or GOF-TLR7/8 (e.g., G325C). Indeed, some variants decrease UNC93B1 stability or receptor interaction while paradoxically increasing signaling output. Together with studies demonstrating that UNC93B1 can facilitate receptor trafficking and activation while also limiting TLR7 signaling and promoting signal termination (15, 16, 25, 27), these observations suggest that the classical GOF/LOF dichotomy oversimplifies the functional consequences of disease-associated UNC93B1 variants.

At pathway level, disease-associated *UNC93B1* variants selectively enhance TLR7 and/or TLR8 signaling, with little or no effect on TLR3 or TLR9 responses (Fig. 1). In THP-1 monocytic cells engineered to express UNC93B1^V117L^ or UNC93B1^T314A^ and in PBMCs from patients, these UNC93B1 variants upregulate TLR7-driven cytokine programs (including responses to the dual TLR7/TLR8 agonist R848), with additional TLR8 agonist hyperresponsiveness in myeloid cells not accompanied by a similar enhancement of responses to TLR3 and TLR9 ligands (48). Similarly, other UNC93B1 variants—including E92G, T93I, R336C, R336H, and R336L—predominantly lead to TLR7-biased signaling accompanied by strong IFN signatures, despite differences in experimental systems between studies (46, 47, 50). The I317M variant, identified in the homozygous state in a patient with SLE, results in enhanced TLR7 signaling and a smaller increase in TLR8 activity in vitro (49), whereas G325C enhances both TLR7 and TLR8 activation, with an increase in UNC93B1–TLR8 association (49). By contrast, TLR8-biased variants, such as L330R, R466S, and R525P, enhance TLR8-dependent inflammatory responses and cytokine production, whereas L330R and R466S also display enhanced UNC93B1–TLR8 association in vitro (49). UNC93B1 levels are high in the peripheral B cells of patients with active SLE and are correlated with IFN signatures, probably reflecting IFN-induced transcription rather than a direct pathogenic contribution (13).

The study of patients with receptor-intrinsic GOF mutations of *TLR7* or *TLR8* provides some insight for the interpretation of UNC93B1-associated phenotypes. X-linked TLR7 GOF mutations cause early-onset autoimmunity, principally SLE, predominantly in female patients, through enhanced TLR7 signaling, excessive type I IFN responses, and B cell activation (60, 61, 62, 63). These disorders are associated with enhanced IFN signatures and dysregulated nucleic acid sensing, features common to several monogenic interferonopathies. By contrast, TLR8 GOF mutations cause neutropenia, antibody deficiency, lymphoproliferation or bone marrow failure, systemic inflammation, and immune dysregulation, often with prominent myeloid involvement and a male predominance (64, 65). Disease-associated UNC93B1 variants broadly reproduce several aspects of this dichotomy. Variants that preferentially amplify TLR7 signaling—including E92G (46), T93I (47), V117L and T314A (48), I317M (49), R336C (47), R336L (46), R336H (50), and E49dup (25)—are associated with phenotypes ranging from very early-onset SLE with strong IFN signatures (E92G) to childhood-onset lupus with nephritis (T314A) or severe autoimmune cytopenias (I317M), consistent with a TLR7-dominant phenotype. Further support for the effects of the human T93I, V117L, and R336C variants has been provided in vivo by knock-in mouse models, which develop systemic inflammatory or lupus-like phenotypes associated with enhanced TLR7-dependent responses (47, 48). By contrast, variants that bias UNC93B1 toward TLR8 activity—including L330R, R466S, R525P (49), and G325C, which enhances both TLR7 and TLR8 signaling but leads to greater UNC93B1–TLR8 association (49)—tend to cause chilblain lupus, skin-predominant disease, pulmonary or systemic inflammation, and prominent myeloid involvement. These phenotypes have selected inflammatory and myeloid features in common with receptor-intrinsic TLR8 GOF syndromes, although the clinical manifestations associated with receptor-intrinsic TLR8 GOF and the UNC93B1-mediated enhancement of TLR8 signaling are different. This pathway-specific clustering may explain why UNC93B1 variants are associated with a spectrum of disease ranging from cutaneous lupus to severe systemic inflammatory disease (49).

## Concluding remarks

UNC93B1 functions as a molecular fulcrum between protective and pathological immunity: germline homozygous LOF variants confer a predisposition to severe viral infection, whereas germline heterozygous or homozygous variants associated with enhanced TLR7 and/or TLR8 signaling confer a predisposition to focal or systemic autoimmunity and hyperinflammation (2, 3, 46, 48, 49) (Fig. 1). This duality illustrates how opposite perturbations of UNC93B1-dependent trafficking and signaling can produce divergent outcomes, from impaired antiviral immunity to systemic IFN-associated inflammation and autoimmunity (24, 25, 40, 45, 46, 47, 48, 49, 66). UNC93B1 thus exemplifies the way in which rare genetic disorders affecting type I IFN immunity can shed light on both fundamental and clinical immunology (67). Other examples of type I IFN–related loci in which different classes of mutations can underlie either viral disease or autoimmunity include TLR7, LOF variants of which confer a predisposition to severe viral infection, with GOF variants causing lupus-spectrum autoimmunity (56, 60, 61, 62, 63, 68, 69, 70), and IFIH1/MDA5, GOF mutations of which cause interferonopathy and autoimmunity, whereas biallelic LOF mutations confer a predisposition to severe viral respiratory infections (71, 72, 73).

The key unresolved questions differ for patients with LOF mutations and signaling-enhancing UNC93B1 variants. Human LOF genotypes impair TLR3 activity in multiple cell types, including dermal fibroblasts, CNS-resident cells, and probably respiratory epithelial cells, together with TLR7, TLR8, and TLR9 activity in leukocytes, including plasmacytoid dendritic cells (pDCs) (3, 4, 11, 22, 40, 51). In this light, the range of viral diseases threatening patients with UNC93B1 deficiency may be broader than currently appreciated. Vulnerability to HSE is firmly established (3, 24), whereas the contribution of heterozygous UNC93B1 variants to severe COVID-19 pneumonia remains to be clarified (45). The contribution of impaired TLR8 and TLR9 signaling to clinical phenotypes also remains unclear. Future discoveries of patients with inherited defects of TLR8 or TLR9 may help delineate the full range of pathogens controlled by UNC93B1-dependent pathways. Moreover, the penetrance, tissue specificity, and interindividual variability of UNC93B1 deficiency for each viral infection require better definition and explanation.

For humans with UNC93B1 variants associated with enhanced TLR7 and/or TLR8 signaling, the quantitative threshold and molecular mechanisms selectively enhancing TLR7 and/or TLR8 signaling remain unclear. Experimental studies have shown that UNC93B1 orchestrates the differential trafficking and signaling of TLR7 and TLR9 (15, 16, 26, 27) and regulates receptor stability (11), but the effects of specific human variants (e.g., TM6–TM7 helix or N-terminal acidic-patch alterations) on these processes remain unclear (25, 46, 47, 48). Two groups of signaling-enhancing UNC93B1 variants result in predominantly dysregulated TLR7 or TLR8 signaling, underlying systemic lupus and chilblain lupus, respectively, but with variable expressivity. The clinical spectrum associated with signaling-enhancing UNC93B1 variants may extend beyond the currently recognized lupus-spectrum and inflammatory phenotypes, as suggested by reports linking UNC93B1 variants to additional inflammatory presentations (44, 50). A more precise definition of phenotypic range, penetrance, and modifying factors is required. Finally, it remains an open question whether TLR3 or TLR9 GOF occurs in humans and whether certain UNC93B1 variants preferentially dysregulate the pathways of these TLRs. Addressing these issues will make it possible to refine our understanding of endosomal nucleic acid sensing and will guide the development of targeted therapeutic strategies.
